# Prohedonic Emotion Regulation Goals in Everyday Life: How Increasing Positive Affect and Reducing Negative Affect Relate to the Emotion Regulation Process and Depression

**DOI:** 10.1007/s42761-026-00375-8

**Published:** 2026-06-08

**Authors:** Alison B. Tuck, Gigi A. Taillon, Renee J. Thompson

**Affiliations:** Department of Psychological and Brain Sciences, Washington University in St. Louis, 1 Brookings Drive, CB 1125, St. Louis, MO 63130 USA

**Keywords:** Emotion regulation, Prohedonic, Depressive symptoms

## Abstract

Research finds that prohedonic emotion regulation (ER) plays a key role in psychological well-being. Despite prohedonic ER encompassing both increasing or maintaining positive affect (PA) and decreasing negative affect (NA), prohedonic ER is often examined without regard to these different forms. Research is needed to examine the frequency of these two forms of prohedonic ER and their associations with depressive symptoms to clarify their costs and benefits. In addition to investigating their frequency, we examined how they were related to perceived ER effort, difficulty, and success and whether depression moderated these associations. Participants (*N* = 179; *M*_age_=35.7) completed an ecological momentary assessment (EMA) protocol where they were surveyed five times a day for 14 days. At each EMA survey, participants reported their emotional responses to the most significant event since the preceding survey, their ER goals, and their ER effort, difficulty, and perceived success. Participants also completed a self-report measure of depressive symptoms. Participants more frequently reported prohedonic ER goals to decrease NA than increase or maintain PA. The ER goal of decreasing NA was associated with higher effort and difficulty (no association with perceived success). The ER goal of increasing or maintaining PA was associated with lower difficulty and higher perceived success (no association with effort). Depressive symptoms only moderated the association between maintaining/increasing PA to predict greater perceived success. These findings suggest unique benefits associated with upregulating PA compared to downregulating NA and underscore the need to differentiate prohedonic PA and NA goals in research and treatment.

**Supplementary Information:**

The online version contains supplementary material available at https://doi.org/10.1007/s42761-026-00375-8.

Emotion regulation (ER)—the process by which individuals manage their emotions in response to internal and external stimuli—plays a crucial role in psychological wellbeing (Nyklíček et al., 2011; Thompson, 1994; Martínez-Priego et al., 2024). Although ample literature examines ER strategy use, or the specific cognitive-behavioral approaches people take to manage their emotions (Martínez-Priego et al., 2024; Boemo et al., 2022), there is significantly less attention to ER goals, or what emotion states they seek while regulating (Gross, 2015). Research has found that people primarily hold prohedonic ER goals (i.e., ER goals to feel better) compared to contrahedonic ER goals (i.e., ER goals to feel worse; e.g., English et al., 2017). Prohedonic ER goals can take different forms: increasing or maintaining positive affect (PA) or decreasing negative affect (NA; Riediger et al., 2009). Although these forms of prohedonic ER goals are critically important for how people regulate to feel better, they are not typically examined individually. To better understand the costs and benefits associated with prohedonic ER, research is needed to examine how prohedonic PA vs. NA ER goals differentially relate to the ER process. In the current investigation, we utilized ecological momentary assessment (EMA) to examine the frequencies of people’s prohedonic ER goals as well as their associations with perceived ER effort, difficulty, and success. We further examined the extent to which depressive symptoms, which are associated with emotion dysregulation (e.g., Liu & Thompson, 2017), moderated the associations between ER goals and perceived ER effort, difficulty, and success.

People most commonly report that they are motivated to feel better, and doing so is associated with high psychological wellbeing (Bloore et al., 2020). However, “feeling better” can be accomplished by either increasing PA or decreasing NA. Although people *both* increase PA and decrease NA, research has focused on how people regulate NA, with relatively minimal research examining PA regulation (for reviews, see Boemo et al., 2022; Fernandes & Tone, 2021; Vanderlind et al., 2020). Further, while ER research has largely emphasized the ER strategies associated with NA and sometimes PA (e.g., Boemo et al., 2022), research into the frequency people have goals to decrease NA versus increase PA and perception of the ER process remains sparse. These could have important research and clinical implications.

There is some indirect evidence that the ER goal to increase PA may have unique benefits compared to the ER goal to decrease NA. Fredrickson’s (2001) Broaden and Build Theory suggests PA broadens people’s perspectives which in turn builds enduring personal resources (e.g., knowledge) and enhances health and fulfillment. This leads to greater PA, and the cycle repeats. The notion that PA can facilitate novel thoughts and behaviors may also explain assertions rooted in cybernetic control theory, which suggests that people have greater cognitive capacity for ER when feeling good compared to feeling bad (e.g., Tamir, 2021). Hence, current literature indirectly suggests that increasing or maintaining PA may be less effortful and difficult and perceived to be more successful than decreasing NA. To clarify the benefits of prohedonic PA regulation, research is needed to directly examine how different forms of prohedonic ER goals are related to these aspects of the ER process.

ER is a dynamic process that includes several related but distinct dimensions (Gross, 2015), including ER effort, difficulty, and success. Although ER can be automatic, it can also require effort (Gyurak et al., 2011). ER effort reflects the amount of energy invested, whereas ER difficulty reflects the perceived challenge of ER (Gross & Jazaieri, 2014). Although ER effort and difficulty are related constructs, they are conceptually distinct. For example, one may exert considerable effort but not find regulation difficult, or vice versa. ER success reflects the effectiveness of an ER episode or attempt at achieving a desired ER goal, and is central to major ER models (e.g., Gross, 2015).

Beyond clarifying the frequency of different prohedonic ER goals, research should examine how indicators of wellbeing shape links between ER goals and the ER process. Individuals suffering from depression might present as good candidates because depression is widely studied and associated with a variety of emotion dysregulation (APA, 2023; Houben et al., 2015; Liu & Thompson, 2017). Although more research is needed in this area, the perceived success of prohedonic ER in individuals experiencing elevated depressive symptoms has been found to exhibit opposite trends to those found in healthy controls, with perception of enhancing PA tending to be more difficult, more effortful, and less successful due to fear of PA (Campbell-Sills & Barlow, 2007). Further, Vanderlind et al. (2022) suggested that people with major depressive disorder disproportionately employ ER strategies that decrease rather than increase PA, contributing to the low PA observed in depression. Despite this compelling argument, research shows people with major depressive disorder do not differ in the extent to which they engage in prohedonic PA versus NA regulation in everyday life (Liu et al., 2023). Additional research with non-clinical samples could clarify how prohedonic goals relate to the ER process with depression as a main consideration, which could provide insights into how prohedonic ER goals relate to wellbeing more broadly.

## Current Study

Using ordinal categorical scales for PA and NA, our first aim was to examine how likely people were to have ER goals to increase or maintain PA versus decrease NA in everyday life (Aim 1). Because NA more often relates to discrepancies in people’s current versus desired emotional states (Tamir, 2021), we hypothesized that people would report more prohedonic NA goals than PA goals (H1). Our second aim was to examine associations between forms of prohedonic ER and the ER process, including perceived ER effort, difficulty, and success (Aim 2). Due to the unique benefits of PA (Fredrickson, 2001; Tamir, 2021), we hypothesized that prohedonic PA goals would be associated with decreased ER difficulty and effort and increased ER success, whereas prohedonic NA goals would be associated with increased ER difficulty and effort and decreased perceived ER success (H2). For significant findings, we conducted follow-up analyses examining whether effects held when accounting for affect evoked by sampled events. Finally, our third aim was to examine the extent to which depressive symptoms moderated the associations between forms of prohedonic ER goals and perceived ER effort, difficulty, and success (Aim 3). We hypothesized that with increasing depression, the associations between ER goals and (a) ER difficulty and (b) ER effort would be more positively correlated, but the associations between ER goals and (c) perceived ER success would be less positively correlated (H3).

### Statements and Declarations

The study’s aims and hypotheses were not pre-registered. This study was part of a larger study that examined adults’ everyday emotions, social media use, goal pursuits, and interoceptive awareness (https://osf.io/7s6wn/). Sample size was determined based on a power analysis for the parent study. Data and analysis scripts have been made openly available at https://osf.io/gpd7k/. The authors have no conflicts of interests to disclose.

## Method

### Participants

The study included 179 adults ages 19 to 63 years (*M*_age_ = 35.7; *SD*_age_ = 12.3). Gender composition was as follows: 85 women (47.48%), 81 men (45.25%), five gender non-binary (2.79%), one agender (0.56%), and seven not reporting (3.91%). Racial demographics were as follows: 120 White (67.04%), 23 Asian (12.85%), 23 Black (12.85%), six Middle Eastern (3.35%), three Indigenous (1.68%), one Native Hawaiian or Pacific Islander (0.56%), three unreported (1.68%). A total of 26 participants identified as Latino/a/x (14.53%). Participants were recruited from the community via flyers, social media advertisements, and through a research participant registry organized by the university hospital system. Participants completed a phone screen to determine eligibility, which included being between the ages of 18 and 65. Due to primary aims for the parent investigation, participants were required to have one active social media profile and could not have health conditions contraindicated for physiology assessment (e.g., certain heart conditions). Eligible participants attended an in-person laboratory session where they completed a series of several computer-based tasks and self-report measures (relevant measures described below). The study was approved by the university’s Human Research Protection Office (IRB 202209063). Participants received $70 for completing the EMA protocol and a $15 bonus for a completion rate of 80% or more.

### Procedures

This study included data from a large study that was focused on understanding adults’ daily emotional experiences. After informed consent, participants completed self-report measures before completing a semi-structured EMA tutorial. Participants downloaded SEMA^3^ (O’Brien et al., 2023), an EMA software, onto their smartphones and completed a practice survey. Beginning the day after the lab session, participants were surveyed five times a day for 14 days. They selected a 15-hour window for surveys, which were randomly distributed across five three-hour periods, with approximately three hours elapsing between each survey on average. Participants had 20 min to complete each survey after the initial prompt with a reminder if they did not complete the survey within 15 min of the prompt. The survey timed out after 20 min had passed and could not be completed. On average, participants completed 69.14% of the surveys (*SD* = 21.52%).

#### Self-Report Measure

**Depressive Symptoms.** Participants completed the Center for Epidemiological Studies-Depression scale, which was developed to assess depressive symptoms in non-clinical samples (CES-D; Radolff, 1977). The 20-item CES-D assesses depressive symptoms over the past week, ranging from 0 (*Never or none of the time (less than 1 day)*) to 3 (*Most or all of the time (5 to 7 days)*). The internal consistency of scale items within the present sample was good (α = 0.88).

#### EMA Measures

**Prohedonic ER Goals.** Participants were asked to consider an event that led to the largest shift in their emotions since the last survey. They responded to two items: “What did you want to do to your NEGATIVE (POSITIVE) emotions in response to this event?” Response options included “increase or maintain them”, “decrease or stop them”, and “I did not want to influence them”. For prohedonic NA goals, a response was coded 1 (versus 0) if “decrease or stop them” was selected for negative emotions, and for prohedonic PA goals, a response was coded 1 (versus 0) if “increase or maintain them” was selected for positive emotions.

**Elicited PA and NA.** Participants considered the event that led to the largest shift in their emotion since the last survey. They responded to the prompts, “to what extent did you feel POSITIVE emotions in response to this event?” (PA) and “to what extent did you feel NEGATIVE emotions in response to this event?” (NA). Items were scored from 1 (*not at all*) to 7 (*extremely*).

**Perceived ER Effort**,** Difficulty**,** and Success.** At each EMA survey, participants completed three items corresponding to perceived ER effort, difficulty, and success in response to the emotion-eliciting event: “In response to this event, how much effort did you put into trying to change or manage your emotions?”, from 1 (*None*) to 7 (*A great deal*), “In response to this event, how difficult was it to try to change or manage your emotions?”, from 1 (*Not at all*) to 7 (*Extremely*), and “In response to this event, how much success did you have trying to change or manage your emotions?”, from 1 (*None*) to 7 (*A great deal*). Each survey received a separate score for perceived ER effort, difficulty, and success.

To assess the construct validity of these items, we administered the NASA Task Load Index (NASA-TLI), which assesses people’s workload associated with completing a task and includes items for difficulty, effort, and success (Hart, 2006), but modified to assess ER. Research shows people’s responses on the NASA-TLI are significant predictors of their self-regulation in performance contexts (e.g., Matthews et al., 2020). As reported in Gross et al. (2025), participants’ EMA reports of ER difficulty, effort, and success were each significantly associated with the corresponding item of task performance from the NASA-TLI, providing support for construct validity (difficulty: *β* = 0.03, *p* =.030; effort: *β* = 0.24, *p* <.001; success: *β* = 0.26, *p* <.001).

### Analytic Plan

All analyses were conducted in R Data Analysis Software (R Core Team, 2024). We used multilevel models (MLMs) to account for the nested structure of the data (i.e., surveys nested within participants). All models included a random intercept for participant ID to account for between-person variability. Continuous predictors (i.e., ER effort, difficulty, and success) were person-mean centered at Level 1 and grand mean centered at Level 2 (i.e., depressive symptoms). The upregulation or maintenance of PA and downregulation of NA were included as fixed effects. Analyses were conducted using the `lme4` package (Bates et al., 2015).

To address **Aim 1**, we compared people’s frequencies of PA and NA prohedonic ER goals by computing a generalized linear MLM predicting both PA and NA goals as two outcome variables in the same model nested within people across surveys. To address **Aim 2**, or to examine how prohedonic goals predicted ER effort, difficulty, and success, we conducted three MLMs predicting (a) ER effort, (b) ER difficulty, and (c) ER success from PA and NA goals entered simultaneously. We also conducted follow-up analyses explicitly isolating instances of PA only regulation, NA only regulation, and both ER goals predicting ER effort, difficulty, and success; we conducted contrast analyses to compare effects. Before testing our final aim, we examined how depressive symptoms related to PA and NA prohedonic ER goals by running two generalized linear MLMs, one predicting (a) PA goals and (b) NA goals from depressive symptoms. For models testing Aims 1 and 2, the binomial family was specified to handle binary outcome variables. Fixed effects from models were extracted to compute odds ratios.

To address **Aim 3**, we examined how depressive symptoms moderated the association between prohedonic ER goals and perceived ER effort, difficulty, and success by running three MLMs predicting (a) ER effort, (b) ER difficulty, and (c) ER success from PA goals, NA goals, depressive symptoms, an interaction between depressive symptoms and PA goals, and an interaction between depressive symptoms and NA goals, entered simultaneously. We also ran follow-up analyses in which depression was measured dichotomously based on participants scoring above or below the clinical cutoff of 16 on the CES-D. For any significant findings, we conducted follow-up analyses in which we ran the same model controlling for group-mean centered PA and NA experienced in response to the event. All beta coefficients were standardized to aid in interpretability.

## Results

The average depressive symptom score in the sample was 13.21 (*SD* = 9.26), which is below the clinical cutoff of 16 (Vilagut et al., 2016). However, the range was zero to 38, with 29.95% of the sample scoring at or above the clinical cutoff, providing substantial variance in depression. Participants’ average PA in response to the ER event was 4.38 out of 7 (*SD* = 0.83), and their average NA was 2.73 (*SD* = 0.81). On average, participants reported one or both types of prohedonic ER goals at 82% (*SD* = 24%) of surveys.

Since people reported both NA and PA prohedonic goals in the majority of surveys (55%), we conducted additional analyses isolating instances in which people reported PA ER goals only, NA ER goals only, or both PA and NA ER goals. Contrast analyses showed that instances of PA only ER goals related to significantly less ER effort and difficulty than instances of NA only ER goals and both ER goals. Instances of NA only ER goals related to significantly less ER success than instances of PA only ER goals and both ER goals. Statistics can be found in Table 2. These analyses lend further support to our claims showing that prohedonic PA ER goals relate to less ER difficulty and effort while prohedonic NA ER goals relate to less success.

### Aim 1

By prohedonic ER type, participants reported only prohedonic NA goals at 17% of surveys (*SD* = 20%), only prohedonic PA goals at 10% of surveys (*SD* = 12%), and both prohedonic ER goals at 55% of surveys (*SD* = 32%). Overall, participants reported prohedonic NA ER goals significantly more frequently than prohedonic PA goals, *β* = 0.08, *SE* = 0.03, OR [95% CI] = 1.08 [1.03–1.15], *p* =.004, consistent with Hypothesis 1.

### Aim 2

Interclass correlations (ICC) for study variables are presented in Table 1. ICCs indicate the percentage of variance accounted for by between-person (versus within-person) differences for each study variable (Merz & Roesch, 2011). The majority of variance was at the within-person level for ER effort, ER difficulty, ER success, PA, and NA. That is, people experienced more variability in study variables at a state-level, with relatively small differences in variability at a trait-level.

**Table 1:** Interclass correlations (ICCs) and within-person correlations for study variables

Variable	ICC	1.	2.	3.	4.	5.	6.
1. ER Effort	0.16	-					
2. ER Difficulty	0.22	**0.51**	-				
3. ER Success	0.25	0.03	**− 0.28**	-			
4. PA	0.21	**− 0.20**	**− 0.38**	**0.32**	-		
5. NA	0.21	**0.33**	**0.49**	**− 0.25**	**− 0.60**	-	
6. Depressive Symptoms	-	0.06	**0.18**	**− 0.18**	**− 0.18**	**0.20**	-

Bolded values indicate significance, *p* <.05. *ER* Emotion regulation, *PA* Positive affect, *NA * Negative affect

Associations between ER goals and ER effort, difficulty, and success are presented in Table 2. Beta comparisons showed all associations between PA and NA goals were significantly different from one another (*p*_*effort*_ < 0.001; *p*_*difficulty*_ < 0.001; *p*_*success*_ = 0.005). Prohedonic NA goals were associated with greater ER effort and difficulty, as hypothesized, but were not significantly related to perceived ER success, inconsistent with our hypothesis. Prohedonic PA goals were unrelated to ER effort, also inconsistent with Hypothesis 2, but were associated with less ER difficulty and greater perceived ER success, as hypothesized. After accounting for emotions experienced in response to the event, prohedonic NA goals were associated with greater ER effort (*β* = 0.15, *SE* = 0.05, *p* =.001) and greater ER difficulty (*β* = 0.14, *SE* = 0.04, *p* <.001), but not success (*β* = 0.05, *SE* = 0.05, *p* =.284). In contrast, prohedonic PA goals were not associated with perceived ER effort (*β* = 0.05, *SE* = 0.05, *p* =.294), difficulty (*β* = 0.06, *SE* = 0.04, *p* =.143) or success (*β* = 0.02, *SE* = 0.04, *p* =.569).

**Table 2:** **A** Emotion Regulation (ER) Effort, Difficulty, and Success Predicted by PA and NA Prohedonic ER Goals Jointly. **B.** Emotion Regulation (ER) Effort, Difficulty, and Success Predicted by PA Only, NA Only, and Both (Reference Group) Prohedonic ER Goals Jointly

	ER Effort		ER Difficulty		ER Success	
	β	*p*	β	*p*	β	*p*
Downregulating NA	0.23	< 0.001	0.27	< 0.001	− 0.01	0.768
Upregulating or Maintaining PA	− 0.03	0.463	− 0.13	0.004	0.17	< 0.001
Downregulating NA Only	− 0.02	0.411	0.08	0.388	− 0.14	0.012
Upregulating or Maintaining PA Only	− 0.31	< 0.001	− 0.34	< 0.001	0.07	0.119
Both Prohedonic ER Goals	0.02	—	0.04	—	− 0.01	—

*PA * Positive affect, *NA * Negative affect, Beta coefficients were standardized

### Aim 3

Depressive symptoms were not associated with the likelihood of people reporting either prohedonic NA goals, *β* = 0.19, *SE* = 0.02, OR [95% CI] = 1.02 [0.99–1.05], *p* =.162, or PA goals, *β* = 0.22, *SE* = 0.02, OR [95% CI] = 1.03 [0.99–1.06], *p* =.14. Moderation analyses showed the main effect of depressive symptoms were significant in predicting more ER difficulty and less ER success (and did not significantly predict ER effort; Table 3). Further, these analyses showed depressive symptoms did not interact with prohedonic NA goals to predict perceived ER effort, difficulty, or success (Table 3). Depressive symptoms also did not interact with prohedonic PA goals to predict ER difficulty or effort, but depressive symptoms strengthened the positive association between PA goals and greater perceived ER success (Table 3). These results were inconsistent with Hypothesis 3 that depressive symptoms would moderate effects of ER goals to predict increased perceived ER effort and difficulty and reduced perceived ER success. Follow-up analyses showed that the same effects held when depression was assessed dichotomously based on scoring above or below the clinical cutoff on the CES-D (Supplemental Materials, Table S1). When accounting for the effect of emotions experienced in response to the event, depressive symptoms interacted with PA goals to predict greater perceived ER success (*β* = 0.15, *SE* = 0.04, *p* <.001).

**Table 3:** Emotion regulation (ER) effort, difficulty and success predicted by depression’s interaction with prohedonic PA and NA ER Goals

Variable	ER Effort		ER Difficulty		ER Success	
	β	*p*	β	*p*	β	*p*
NA Goals	0.09	0.003	0.10	0.001	0.00	0.876
PA Goals	− 0.01	0.571	− 0.05	0.964	0.07	0.546
Depression	0.06	0.081	0.17	< 0.001	− 0.18	< 0.001
NA Goals x Depression	− 0.02	0.731	0.00	0.978	0.00	0.994
PA Goals x Depression	− 0.05	0.252	− 0.04	0.063	0.14	0.002

*PA * Positive affect, *NA * Negative affect, NA goals refer to decreasing NA; PA goals refer to maintaining or increasing PA

## Discussion

When people engage in ER, they most typically engage prohedonic ER (Bloore et al., 2020). Prohedonic ER includes increasing/maintaining PA and decreasing NA. Using EMA, we examined how often people pursued prohedonic ER goals, their perceived effort, difficulty, and success, and whether depression moderated these associations.

Consistent with our hypotheses, people more frequently reported decreasing NA than increasing or maintaining PA. Despite this, although decreasing NA goals was reported more frequently, it was related to more ER difficulty and effort (but not perceived success), whereas increasing PA goals related to less difficulty (but not effort) and greater perceived ER success. Together, our results appear to provide empirical support for the Broaden and Build Theory of PA (Fredrickson, 2001) by illustrating that increasing or maintaining PA may have unique benefits with regard to ER difficulty and success compared to decreasing NA.

To examine how wellbeing was implicated in people’s prohedonic ER goals, we also examined effects by depressive symptoms. Depressive symptoms did not relate to the likelihood of people’s prohedonic PA or NA goals, but greater depressive symptoms related to more ER difficulty and less ER success. Further, depressive symptoms did not moderate the effects of prohedonic NA goals to predict perceived ER effort, difficulty, and success. However, although depressive symptoms were associated with less ER success overall, greater depressive symptoms were associated with greater perceived ER success from PA ER goals. Although replication is needed, these findings suggest that those with elevated depressive symptoms benefit most from pursuing prohedonic PA ER goals. These findings appear consistent with the mood-brightening effect, a phenomena wherein people with depression show stronger positive reactivity despite reporting more negative experiences (e.g., Bylsma et al., 2011; Khazanov et al., 2019). These findings challenge proposals in the literature that people with depression *want* to feel bad, often as a means of self-verification (e.g., Arens & Stangier, 2020). If true, we would have expected greater depressive symptoms to relate to less frequent prohedonic ER goals and less effort put towards pursuing prohedonic ER goals. This said, future research should test these moderation effects in a larger, more well-powered sample. It is possible that if moderation effect sizes were small, larger samples will be needed to detect them. In addition, future research should directly examine the perceived ER effort, difficulty, and success of prohedonic ER goals in a clinically depressed sample, where the effect sizes of emotional challenges associated with depressive symptoms may be larger and more salient.

When we accounted for emotional reactions to events, some effects held, and others were no longer significant. Specifically, prohedonic NA goals were still associated with greater ER effort and difficulty, suggesting emotion experienced in response to the ER-eliciting event did not account for the links between NA goals and perceptions of the ER process. However, prohedonic PA goals were no longer associated with ER difficulty or success. These findings might suggest that emotions evoked by the event itself were stronger predictors of ER difficulty and success than the PA goals themselves, at least when examined concurrently. Since emotions tend to result from people’s ER goals (Martínez-Priego et al., 2024), it is challenging to parse apart emotions from their ER goals; hence these findings make sense. Further, they do not necessarily detract from our findings that when people held prohedonic PA goals, they tended to experience less ER difficulty and more ER success. Importantly, this finding does not undermine the value of PA goals, but does suggest a more complex way of understanding PA goals; PA goals may depend more heavily on the affective context for their effects to emerge. Future research should parse apart prohedonic PA goals (i.e. to maintain PA vs. upregulate PA) to directly test whether there are differences in terms of ER effort, difficulty, and success. For example, it is possible that maintaining PA requires less effort, is less difficult, and is perceived to be more successful than upregulating PA. This is an empirical question that needs testing as future research continues to parse apart the nuances of prohedonic ER.

These findings have important research and clinical implications. Researchers interested in the nuances of how people regulate their emotions to feel better should consider differentiating between the upregulation of PA and the downregulation of NA, since these goals differentially relate to ER success. Hence, simply asking participants how they regulate their emotions to feel better may not capture important nuances on how their specific ER goals relate to outcomes. From a clinical perspective, much psychotherapy centers on helping patients decrease NA rather than increase PA. For instance, cognitive behavioral therapy, the most efficacious treatment for depression and anxiety (Barlow, 2004), largely focuses on addressing negative thinking processes and maladaptive behaviors with the goal of reducing negative affective experiences (Craske et al., 2019). This may help explain why cognitive behavior therapy predicts greater decreases in NA over time compared to increases in PA (Whelen & Strunk, 2021). However, the present findings that increasing PA was less difficult and perceived to be more successful, especially with increasing depressive symptoms, suggest that psychotherapy may benefit from greater efforts towards helping patients increase PA, assuming these findings also characterize clinical samples. These interpretations of potential benefits associated with PA upregulation in psychotherapy should also be considered in the context of research showing that efforts to increase positive emotions or “feel good” can sometimes backfire or have unintended negative consequences. For example, a recent review of the literature concluded that pleasant emotions can be costly for the individual if experienced in situations in which the pleasant emotion is not functional (e.g., in confrontational scenarios; if there is a threat in the environment; Ford, 2025). It will therefore be important for therapists to not over-value positive emotion in therapy, but rather emphasize working towards PA goals when situationally appropriate. At the same time, future research could directly compare treatment outcomes between patients whose treatment largely focused on addressing NA reduction compared to PA enhancement, such as in behavioral activation (Mazzucchelli et al., 2010). In addition, future research should extend this work by examining the specific strategies people use to pursue their ER goals. For example, one might not intend to downregulate NA as a goal, but nonetheless find using the strategy of reappraisal easier to engage than acceptance, or vice versa. Likewise, some strategies may be less effortful or more successful depending on different contexts (e.g., being alone versus with others). Future research should examine how particular ER strategies align with people’s regulation goals and perceived effort, difficulty, and success, as this may clarify the processes that underlie effective ER in daily life.

The current investigation focused on better understanding prohedonic ER goals. Although people were more likely to decrease their NA in everyday life contexts, increasing or maintaining PA was less difficult and resulted in greater perceived success, whereas decreasing NA related to more ER effort and difficulty. These findings clarify how people regulate their emotions to feel more good versus less bad, the most common ER goals (e.g., English et al., 2017) that are rarely examined separately. Findings provide support of the benefits associated with upregulating PA that were not associated with downregulating NA, which may have important clinical implications.

## Supplementary Information

Below is the link to the electronic supplementary material.

Supplementary material 1 (DOCX 16.5 KB)

## Additional Information

### Data Availability

Data and analysis scripts have been made openly available at https://osf.io/gpd7k/.
