# Supplementary material for: Prohedonic Emotion Regulation Goals in Everyday Life: How Increasing Positive Affect and Reducing Negative Affect Relate to the Emotion Regulation Process and Depression
Source: Affect Sci. 2026 Jun 8;7(3):523–30. doi: 10.1007/s42761-026-00375-8 (PMC13582696; doi:10.1007/s42761-026-00375-8)
Supplement: Supplementary file 1 [file 42761_2026_375_MOESM1_ESM.docx]

**Supplemental Materials**

**Table S1**

*Emotion Regulation (ER) Effort, Difficulty and Success Predicted by Depression’s Interaction with Prohedonic PA and NA ER Goals when Depression is Assessed as Either Above or Below Clinical Cutoff*

| **Variable** | **ER Effort** | | |  | **ER Difficulty** | | |  | **ER Success** | | |
| --- | --- | --- | --- | --- | --- | --- | --- | --- | --- | --- | --- |
|  | *β* |  | *p* |  | *β* |  | *p* |  | *β* |  | *p* |
| **NA Goals** | .24 |  | <.001 |  | .26 |  | <.001 |  | .00 |  | .990 |
| **PA Goals** | .01 |  | .813 |  | -.06 |  | .321 |  | .06 |  | .278 |
| **Depression** | .18 |  | .180 |  | .36 |  | .008 |  | -.40 |  | .004 |
| **NA Goals x Depression** | -.01 |  | .894 |  | .03 |  | .797 |  | -.04 |  | .670 |
| **PA Goals x Depression** | -.12 |  | .202 |  | -.17 |  | .061 |  | .25 |  | .005 |

*Note.* PA = Positive affect; NA = Negative affect; NA goals refer to decreasing NA; PA goals refer to maintaining or increasing PA
